# Nutritionally Improved Wheat Bread Supplemented with Quinoa Flour of Large, Medium and Small Particle Sizes at Typical Doses

**DOI:** 10.3390/plants12040698

**Published:** 2023-02-04

**Authors:** Ionica Coţovanu, Costel Mironeasa, Silvia Mironeasa

**Affiliations:** 1 Faculty of Food Engineering, Stefan cel Mare University of Suceava, 13 Universitatii Street, 720229 Suceava, Romania; 2 Faculty of Mechanical Engineering, Automotive and Robotics, Stefan cel Mare University of Suceava, 13 Universitatii Street, 720229 Suceava, Romania

**Keywords:** wheat–quinoa composite flour, amino acids, minerals, rheological properties, bread, nutritional profile, sensory characteristics

## Abstract

One of the food industry’s challenges is to enhance bread quality from a nutritional point of view without impacting negatively sensorial characteristics and consumer decisions on product choice. This study aimed to assess the baking characteristics of wheat bread supplemented with quinoa flour (QF) of large, medium and small particle sizes at typical doses previously established based on an optimization process, and to evaluate the optimal bread from a physical, textural, nutritional, and sensorial point of view. The results showed a decrease in the Falling number index, water absorption, dough stability, speed of protein weakening, dough extensibility, and creep-recovery compliances for optimal wheat–quinoa composite samples with large and medium particle sizes; meanwhile, for the samples with small particle sizes an opposite trend was recorded, with the exception of dough extensibility. Dough fermentation parameters and bread volume rose for all optimal formulations, while firmness decreased compared to wheat bread. All optimal bread samples presented an improved nutritional profile depending on the particle size. The protein content was up to 19% higher, ash up to 13.8%, and lipids up to fifteen times higher. A noticeable enrichment in minerals (mainly K, Mg, Na, Zn, up to 2.3 times) and essential amino acids (with 13.53%) was also obtained for all optimal breads. From an acceptability point of view, the highest score (8.70) was recorded for the optimal bread with a QF of medium particle size. These findings offer processors new information which will be useful for diversifying bakery products with an enhanced nutritional profile.

## 1. Introduction

Cereals and cereal products have been the major component of the human diet throughout the world since ancient times. Wheat represents one of the most important cereals in terms of human nutrition, being from a nutritional point of view an important source of complex carbohydrates, group B vitamins, iron, and trace minerals, especially calcium, phosphorus, iron, potassium, and dietary fibers, with low lipid content [1]. However, wheat is not consumed directly as a grain; it is ground into flour—which changes its nutritional profile, especially crude fiber—followed by ash [2]. Wheat is a unique grain that is suitable for the preparation of a wide range of fermented bakery products to meet the demands of consumers worldwide [1]. Among these products, bread has been a staple food for many civilizations, and is the base of the food pyramid. 

Bread has a fundamental role in nutrition due to the adequate balance of macronutrients in its composition; in addition, it offers some minerals and micronutrients. White bread, available in most global markets, has a low content of nutrients due to the use of refined wheat flour as a raw material for its manufacture. Nowadays, consumers are more and more interested in their health, and buy foods with beneficial effects on health; to accommodate this, the baking industry is trying to provide functional food with pro-health properties. At the same time, there is a need to develop bakery products with new organoleptic properties which meet the needs of the market. The use of composite flour for the manufacture of bread has gradually gained importance for various economic and nutritional reasons. Different studies have been carried out on the influence of wheat–pseudocereal composite flour on the physico-chemical properties of bakery products, and their relationship with sensory properties and nutritional advantages [3,4,5]. Additionally, in the bakery industry, there are ongoing studies on expanding the nutritional value and sensorial acceptability of wheat breads with various added plant-based ingredients [6,7,8].

An enrichment of wheat flour with minerals, and implicitly, an increase in the biological value of bread, can be achieved by adding quinoa flour. Wang et al. [9] studied the use of quinoa flour in baked goods as an additive to wheat flour and concluded that addition doses of 15–75% produced significant effects in bread and cakes. Some studies [10,11] showed that the addition of quinoa flour to wheat flour led to an improvement in bread quality due to the lipids from quinoa flour. Compared to wheat flour products, those made from wheat–quinoa composite flour had a lower specific volume, firmness, chewiness, and a darker color [12]. However, the bread with quinoa flour was well accepted, with an overall score of 5.8 (on a 7-point hedonic scale), and 85% of consumers surveyed said they would buy the product because they liked the taste, and also because of the health benefits [12]. In another study [13], sensory evaluation of bread prepared with whole quinoa flour at a substitution level of 25% indicated a score that was not substantially different from wheat flour bread. Instead, for the bread with 50% quinoa flour, consumers identified a denser and more compact crumb compared to wheat flour bread, but this product was also accepted by consumers [13]. Some researchers have identified that quinoa flour gives bread a nutty flavour and crunchy texture [14]. According to the results reported by different groups of researchers, whole quinoa flour could be a good substitute for wheat flour in bread formulations that aim to increase the nutritional value of the product with dietary fiber, minerals, proteins and healthy fats, with only a small reduction of bread quality [13,15]. For bread containing 10% quinoa flour, similar scores to those for the control sample were obtained in terms of the sensory profile [16]. The use of quinoa flour during the baking process led to the breakdown of simple polyphenols present in quinoa seeds (protocatechuic acid and vanillic acid derivatives), while flavonoids such as quercetin and kaempferol glycoside were preserved [17], and have multiple beneficial effects on health [18]. An improvement in the content of iron, potassium, magnesium, manganese, and zinc was reported for bread with added quinoa flour, and this correlated with the dose used [13,16]. The incorporation of quinoa flour into bread could also be an area that can be exploited to affect eating behavior/appetite control [19]. 

Various studies have revealed quinoa flour’s potential to improve the rheological behavior of wheat flour dough and the quality of finite products. However, there is a lack of reports on the effects of the optimal wheat–quinoa composite flour of three different QF particle sizes on the rheological properties of dough and the features of bread. Thus, the purpose of this work is to ascertain the effect of optimal composite wheat–quinoa flour of large, medium and, small QF particle sizes on bread quality parameters in terms of chemical, physical, textural and sensory characteristics. This study could shed new light on aspects of the practical use of different quinoa particle sizes to substitute wheat flour.

## 2. Results

### 2.1. Chemical Composition of Optimal Wheat–Quinoa Composite Flours Versus Wheat Flour 

**Figure 1:**
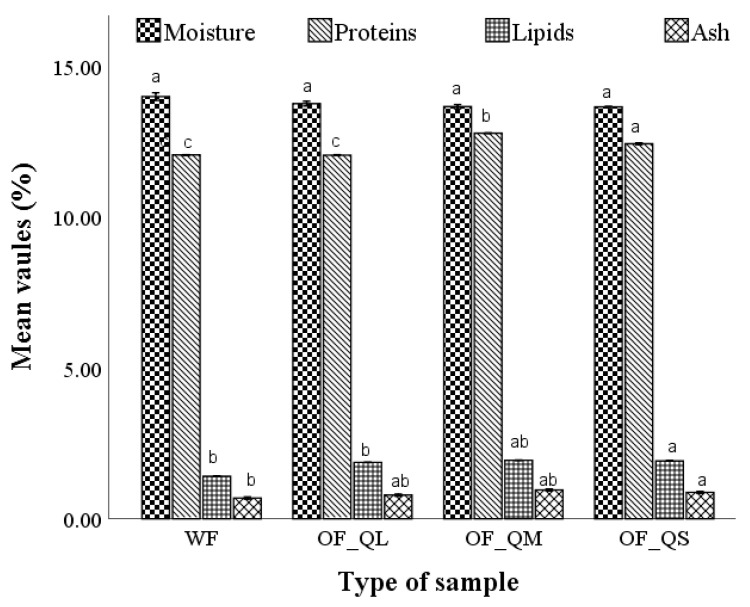
Chemical composition of wheat flour (WF) and optimal wheat–quinoa composite flour with large (OF_QL), medium (OF_QM) and small (OF_QS) particle size. Mean values followed by different letters (a–c) are significantly different (*p* < 0.05).

The distribution of chemical components of optimal wheat–quinoa composite flours with large, medium, and small particle sizes (OF_QL, OF_QM, and OF_QS) compared to wheat flour (WF) is shown in Figure 1.

The optimal wheat–quinoa composite flours did not show significant differences (*p* > 0.05) regarding moisture (Figure 1). The protein content of optimal composite flours varied from 12.41 to 13.13% depending on the quinoa flour particle size, and a significant difference (*p* < 0.05) was found between wheat flour and optimal composite flours with small and medium QF particle sizes (Figure 1). The lipid content of optimal composite flours (1.87–1.94%) was significantly (*p* < 0.05) higher than the lipid content of wheat flour (1.40%). A remarkable difference was found also between the ash content of the optimal composite flours (0.85–0.95%) and wheat flour (0.65%). 

The results regarding the macro- and micro-elements of optimal wheat–quinoa composite flour vs. wheat flour were presented in Table 1. A notable difference in the share of potassium (K) among the tested flours was observed in wheat flour (108.5 mg/100 g), quinoa flour (243.50 mg/100 g), and the optimal composite flour with large quinoa flour particle size (135.70 mg/100 g); meanwhile, the optimal composite flours with medium and small particle sizes presented the smallest values. Magnesium is the richest element in the studied flours (Table 1). Additionally, significant differences (*p* < 0.05) were observed regarding iron content for all studied flours, varying between 1.80–8.12%. Regarding the flours’ zinc content, there were no significant differences between samples. 

The variation in macro- and micro-elements showed an increased content for the optimal wheat–quinoa composite flour corresponding to the large, medium and small particle sizes of quinoa flour compared to wheat flour, and the highest values were found in composite flour with large particle size (OF_QL).

**Figure 2:**
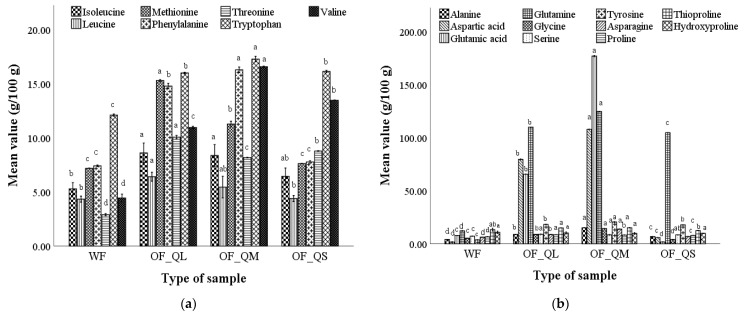
Essential (**a**) and non-essential (**b**) amino acid content of optimal wheat–quinoa composite flour corresponding to large, medium, and small particle size (OF_QL, OF_QM, and OF_QS) vs. wheat flour (WF). Mean values followed by different letters (a–d) are significantly different (*p* < 0.05).

The amino acid content of the optimal wheat–quinoa composite flour compared to that of wheat flour is shown in Figure 2.

The optimal wheat–quinoa composite flour presented considerable higher amounts for essential amino acids isoleucine (5.69–5.91%), methionine (7.23–7.92), phenylalanine (7.41–8.32%), tryptophan (12.39–12.59%) (Figure 2a), and non-essential amino acids aspartic acid (1.98–12.77%), glutamic acid (7.34–25.78%), and glutamine (21.23–24.19%) (Figure 2b), compared to wheat flour, which contains 5.57% isoleucine, 7.19% methionine, 7.37% phenylalanine, 12.03% essential amino acids and 1.54% aspartic acid, 7.97% glutamic acid, and 12.33% glutamine. It can be observed that when quinoa flour particle sizes decreased in the optimal composite flour, the amino acid content increased significantly, especially for essential amino acids. In optimal wheat–quinoa composite flours with medium and small particle sizes, the highest content of methionine, phenylalanine, tryptophan, glutamine, serine, asparagine, and proline was found. The increase in amino acid content in the composite flours with medium and small particle sizes can be compared to the highest content of quinoa flour of the composite flour with large particle size.

### 2.2. Evaluating the Baking Characteristics of the Optimal Wheat–Quinoa Composite Flour and the Quality of the Bread

The baking characteristics of the optimal wheat–quinoa composite flour for each quinoa flour particle size achieved by using different devices (Falling Number, Mixolab, Alveograph, Rheofermentometer and Dynamic Rheometer) in order to make a complete evaluation are shown in Table 2.

The formulation of wheat–quinoa composite flours with the optimal addition doses typical of each QF particle size aimed to obtain the best technological and quality characteristics in bread. The results indicated a slight increase in the Falling number index in the optimal composite flour with small particles when compared to the wheat flour. Water absorption capacity and C1-2 and C5-4 torques indicated a slight decrease for optimal wheat–quinoa flour with large and medium particle sizes, while dough development time and C3-4 torque raised comparatively with values registered for wheat flour. 

Alveographic parameters indicated an increase in tenacity and alveographic P/L ratio for all the optimal composite flour, while extensibility and deformation energy decreased compared to the control. 

The fermentation behavior of the composite flour doughs indicated an improvement in terms of gas formation and retention capacity compared to the wheat flour dough. 

The elastic and viscous moduli of the optimal wheat–quinoa dough showed higher values, while the viscosity factor (tan δ) and the maximum gelatinization temperature (T_max_) indicated a substantial decrease compared to wheat flour (Table 2). A higher resistance to deformation indicated by the decrease of the creep-recovery compliance value was obtained for the optimal samples with large and medium quinoa particle sizes compared to the wheat flour dough. In the case of samples with small particle sizes of quinoa flour, the resistance to deformation is close to that of the wheat flour dough. 

### 2.3. Advanced Characterization of the Bread Made from Optimal Wheat–Quinoa Composite Flour Typical to Each Particle Size

#### 2.3.1. The Physical Characteristics of Bread Obtained from the Optimal Composite Flour for Each Quinoa Flour Particle Size Studied 

The physical characteristics, volume, specific volume, porosity, and elasticity of the bread obtained are presented in Table 3, showing significant differences (*p* < 0.05) between the samples. The appearance and section of the bread samples with the optimal dose of quinoa flour corresponding to the large, medium, and small particle size (OB_QL, OB_QM, and OB_QS) compared to wheat flour bread are shown in the Supplementary Material (Figure S1).

For the bread samples made from optimal composite flour, all the particle sizes which substitute wheat flour at typical doses improved the samples volume, specific volume, porosity, and elasticity (Table 3). The best results in terms of technological parameters were obtained for the sample with large quinoa flour particle size. 

The color parameters of the optimal bread crust were considerably influenced by the particle size, the crust lightness of the wheat flour bread being much higher than that of the optimal bread crust. The lightness of the optimal bread samples decreased with decreasing particle size (Table 4).

The crumb lightness (L*) of the bread samples also decreased depending on particle size, and the lowest values was found for optimal bread with medium QF particles. The variation of redness (a*) and yellowish (b*) color parameters showed an irregular trend (Table 4). The total color difference (ΔE) values of bread crust and crumb increased with the decrease in QF particle size compared to wheat flour bread, which indicates that color variation could easily be perceived without a closer visual inspection [7].

#### 2.3.2. Assessing the Texture Parameters of Optimal Bread

The firmness, springiness, gumminess, and masticability significantly (*p* < 0.05) decreased in the optimal wheat–quinoa composite bread compared to wheat flour bread, while the cohesiveness did not present considerable differences between the bread samples, even though a slight decrease was observed with the increase in particle size. (Table 5). Resilience increased significantly for the optimal bread obtained with medium and small quinoa flour particle sizes, whereas the bread with a large quinoa flour particle size presented the lowest value for this parameter in comparison with wheat flour bread. The optimal composite flour bread crumb firmness decreased significantly (*p* < 0.05) compared to the control sample (5.71 N), the highest value being found in bread with large quinoa flour, while the lowest value was obtained for the bread with small quinoa flour particle size (4.39 N). The highest values of crumb masticability after wheat flour-based bread are offered by the bread with a medium quinoa flour particle size. Evaluation of the optimal wheat–quinoa flour typical to each particle size’s effect on the bread textural parameters is valuable because it indicates consumers’ perception when they chew the bread.

#### 2.3.3. Nutritional Composition and Energetic Value of Optimal Breads 

The physicochemical properties of the bread made from the optimal composite flours for each quinoa particle size compared to the wheat bread are shown in Table 6.

The proximate composition of the optimal bread samples, corresponding to each quinoa flour particle size, showed significant differences (*p* < 0.05) among themselves and compared to wheat flour. The moisture content of the optimal bread presented a slight increase, especially in the optimal bread with medium particle size (Table 6). Moreover, an increase in the protein content, especially for the optimal bread with medium and small quinoa flour particle size, was observed. The lipid and ash contents of bread showed higher values in the optimal composite flour bread, especially in the case of bread with medium and small particle sizes of quinoa flour, while the carbohydrate content and energy values decreased considerably in the bread with medium quinoa particle size.

#### 2.3.4. The Amino Acid Content of Optimal Bread Samples

**Figure 3:**
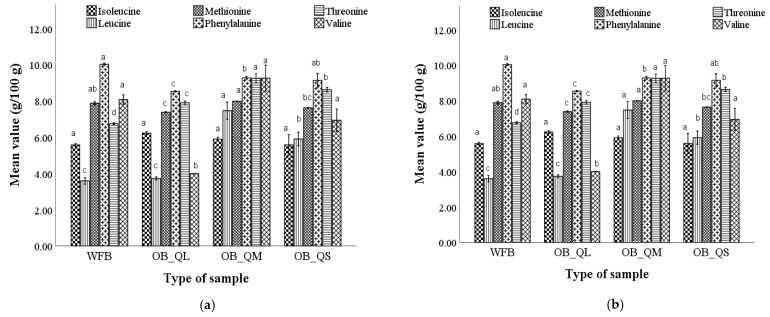
Essential (**a**) and non-essential (**b**) amino acid composition of the bread with the optimal dose of quinoa flour corresponding to the large, medium, and small particle size (OB_QL, OB_QM, and OB_QS) compared to wheat flour bread (WFB). Mean values followed by different letters (a–d) are significantly different (*p* < 0.05).

The variation of essential and non-essential amino acid (AA) content in optimal bread samples corresponding to large, medium, and small particle size is presented in Figure 3. We can observe from Figure 3a that six essential AA were identified in optimal breads, and represent 15.53% for the sample with large QF particles (OB_QL), 14.80% for the sample with medium particles (OB_QM) and 14.00% for the sample with small particles (OB_QS) of the total AA content. Regarding the AA profile, in optimal bread, eleven non-essential amino acids were also identified (Figure 3b). 

The amino acid content of the bread samples obtained from the optimal wheat–quinoa composite flour varied as follows: isoleucine (5.58–6.23%), leucine (3.73–7.47%), methionine (7.38–7.94%), phenylalanine (8.54–9.28%), threonine (7.90–9.25%), valine (3.99–9.26%), alanine (5.36–5.87%), aspartic acid (24.56–43.28%), glutamic acid (10.83–55.45%), glutamine (108.26–128.24%), glycine (2.79–12.48 %), serine (6.37–7.05%), tyrosine (10.05–11.30%), asparagine (6.53–6.78%), proline (7.56–8.01%), thioproline (12.49–20.69%), hydroxyproline (9.60–14.26%). According to the data presented, the wheat flour bread showed significantly lower amounts of essential amino acids (leucine, methionine, threonine) compared to the optimal bread with quinoa flour particle sizes. The bread obtained from composite flour with medium and small quinoa particle sizes presented generally higher levels of essential amino acids compared to the bread with the large quinoa particle size. Generally, among the non-essential amino acids, the highest amounts were found for aspartic and glutamic acid, and thioproline. The highest amounts of aspartic acid and hydroxyproline were recorded in the optimal bread with small particle size, and the highest amount of serine was recorded in the optimal bread with medium particle size.

#### 2.3.5. Determination of Macro- and Micro-Element Content of Optimal Bread Samples

**Figure 4:**
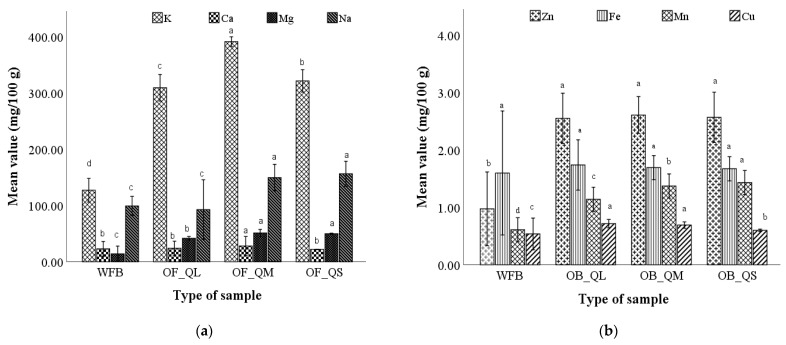
Macro–(**a**) and micro-mineral (**b**) composition of the bread with the optimal dose of quinoa flour corresponding to the large, medium, and small particle size (OB_QL, OB_QM, and OB_QS) in comparison with wheat flour bread (WFB). Mean values followed by different letters (a–d) are significantly different (*p* < 0.05).

The macro- and micro-element content of the bread samples obtained from the optimal wheat–quinoa composite flours showed variation depending on quinoa particle size composition (Figure 4).

For bread made from optimal wheat–quinoa composite flour, an increase in mineral content was obtained in the sample with medium-sized particles, followed by the sample with small-sized particles, and then by the one with large-sized particles. 

#### 2.3.6. Sensory Analysis of Bread from Optimal Wheat–Quinoa Composite Flours

**Figure 5:**
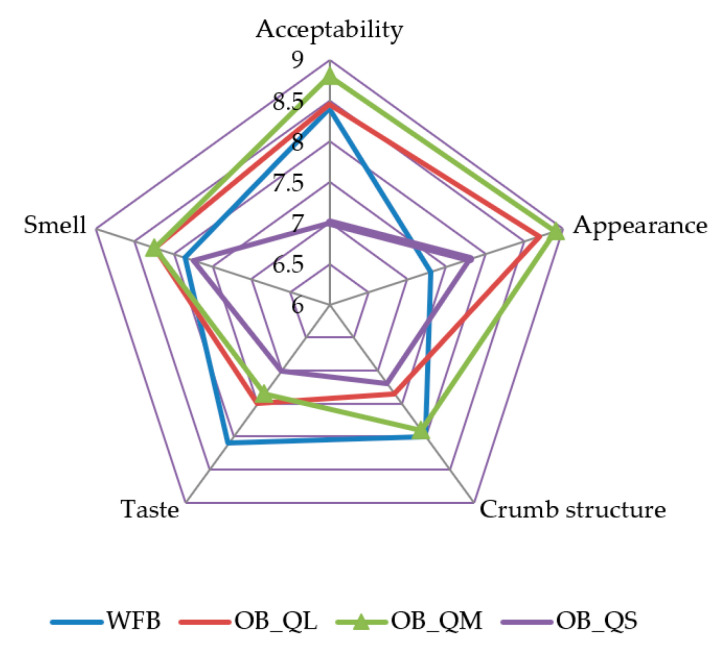
Sensory characteristics of bread samples with the optimal dose of quinoa flour addition corresponding to the large, medium, and small particle size (OB_QL, OB_QM, and OB_QS) versus wheat flour bread (WFB).

The results of the sensory analysis revealed some improvements in terms of overall acceptability, appearance, core structure, taste, and smell of the bread samples made from the optimal composite flours containing medium and large quinoa flour particles, compared to the wheat flour bread (Figure 5).

From the all optimal breads, the bread with the small particle size of quinoa flour was the least preferred, registering a lower score for overall acceptability (Figure 5).

### 2.4. Assessing Relationships between Variables

**Figure 6:**
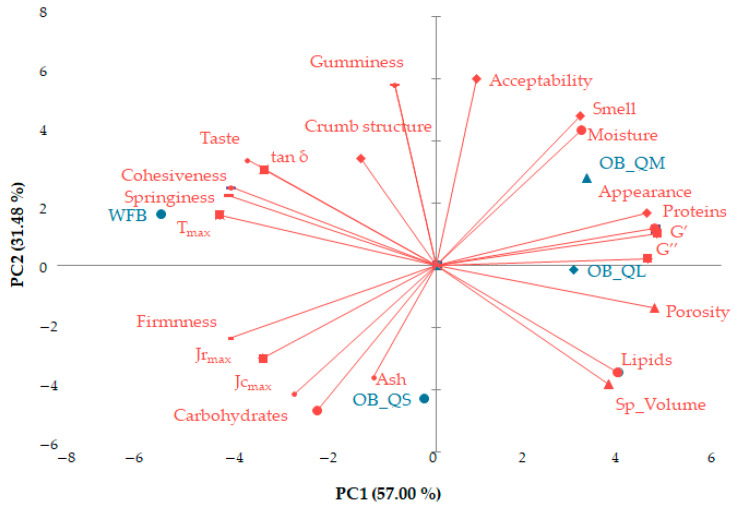
Principal component bi-plot analysis highlighting the relationships between dynamic dough rheological parameters and bread physical-chemical, textural and sensory characteristics. WFB—Wheat flour bread; OB_QL—Optimal bread with large particle size quinoa flour; OB_QM—Optimal bread with medium particle size quinoa flour; OB_QS—Optimal bread with small particle size quinoa flour.

In order to demonstrate comprehensively the similarities and/or differences between the evaluated variables, a multivariate technique, principal components analysis (PCA), was applied. The relationships between dough rheological parameters, bread proximate composition, physical, textural and sensorial characteristics in the bread samples formulated are shown in Figure 6. The first two components PC1 and PC2 explain over 88 % of the total variance. Obvious differences between the optimal bread with large and medium particle size (OB_QL and OB_QM) and wheat flour bread (WFB), explained by PC1, has resulted. PC2 underlines a clear separation between the optimal bread with quinoa flour of medium particle size (OB_QM) and the bread with quinoa flour of small particle size (OB_QS).

## 3. Discussion

### 3.1. Chemical Characterization of Optimal Wheat–Quinoa Composite Flours Versus Wheat Flour

The variation of the optimal wheat–quinoa composite flour protein content was in accordance with the results obtained by other authors [20,21]. This variation can be explained by the locations of the proteins, which are mainly found in the embryo (23.5%), while only 7.2% are found in the perisperm. D’Amico et al. [22] identified higher amounts of protein, up to 38% in the embryo, and less than 5% in the perisperm. A low protein content located in the perisperm was also confirmed by Lindeboom et al. [23]. Other researchers reported a directly proportional increase in protein content with decreasing particle size [24]. Increasing ash content with decreasing particle size of quinoa flour was also observed by Ahmed et al. [25], and Alonso-Miravalles and O’Mahony [26]. Minerals such as phosphorus, potassium, and magnesium are located in the embryo, while the calcium in the pericarp is associated with those pectic compounds of the cell wall [27]. Potassium was present in the highest amounts in all optimal flour samples, and varied between 116.00 and 135.70 mg/100 g, the highest values being observed in OF_QL. For the OF_QS, which consists mainly of the endosperm part of the seed, a large amount of nutritionally important minerals was obtained. Similar results were reported in some studies [28,29,30]. Regarding the amino acids content, the results obtained are similar to those reported by some researchers who evaluated the amino acid content of quinoa flour [28,29,30].

### 3.2. The Baking Characteristics of the Optimal Wheat–Quinoa Composite Flour and the Quality of the Bread

The variation in the Falling number index for optimal composite flour is related to the quinoa starch. Despite the fact that the specific surface area of quinoa starch is higher than wheat starch, it is more sensitive to α-amylase hydrolysis than wheat starch [31]. Additionally, quinoa presents a lower amylase activity, which can increase the production of gas and thus the volume of bread [32]. Other studies [32,33] found similar data on the technological parameters of the bread and cakes with the addition of different quinoa particle sizes, with the bread volume decreasing with the reduction in the particle size. In this research, a rise of all reofermentometric parameters for wheat–quinoa composite flour dough was registered; this is due to fermentable sugars from quinoa flour and the indirect bread-making method. 

The addition of quinoa flour of medium and small particle size to the composite flour led to a firmer dough, which was possibly due to the high protein content of the smallest quinoa flour particle sizes. In bread in which a quantity of wheat flour is replaced by gluten-free flour, the dough viscosity before the starch gelatinization is decisive in preventing the sedimentation of the flour particles so that the gas cells grow and thus maintain a homogeneous system during the fermentation and baking process until starch gelatinization [34,35,36]. The result obtained for the bread volume and crumb elasticity and porosity suggests that lipids from quinoa can act as surface active agents and thus contribute to the stabilization of gas cells before starch gelatinization [34]. The lipid content of quinoa seeds has been reported to be 2–3 times higher than that of buckwheat or common grains such as wheat [37,38]. The polar lipid content of quinoa seeds is very high, and accounts for about 25% of total lipids [37,38]. Thus, the high level of polar lipids in quinoa seeds may play a role in stabilizing gas cells during bread making. The use of emulsifiers in baking has been shown to have a positive effect on the kernel [39,40]. Fatty acids in lipids, such as monoglycerides, can form complexes with amylose, thus limiting starch swelling during baking. As a result, fewer complex substances will form between starch granules and amylose, leading to bread with a softer crumb structure [34,41]. This effect supports the hypothesis that emulsifiers naturally present in quinoa flour can have a positive effect, resulting in bread with a softer and more elastic crumb.

### 3.3. Advanced Characterization of the Bread Made from Optimal Wheat–Quinoa Composite Flour Typical to Each Particle Size

For the bread made from optimal composite flour, the best results in terms of technological parameters were obtained for the optimal bread with a large particle size, but all fractions positively influenced the volume of the samples, being significantly higher than the wheat bread volume. Bread volume and crumb structure depend on a number of factors, such as dough viscosity, amylose/amylopectin ratio, presence of surfactants, and/or protein denaturation (hydrolysis) upon heating [34,35]. The volume increase can be related to the high lipid content of the optimal composite flour with large and medium quinoa particle sizes (Figure 1), which can act as a surface active agent and thus contribute to the stabilization of gas cells before starch gelatinization [34]. The lipid content of quinoa seeds has been reported to be 2–3 times higher than that of common cereals such as wheat [18].

The partial replacement of wheat flour with quinoa flour produced redder, yellower, and lighter bread as indicated by the ∆E values. The high ∆E values of optimal wheat–quinoa bread indicate that color changes could easily be perceived without closer visual inspection, in comparison to wheat flour bread. The decrease in the lightness of crust and crumb of the bread can be explained by Maillard reaction products which are influenced by the distribution of water and the reaction between reducing sugars and amino acids. Carotenoids, chlorophyll, and lignin from quinoa seeds influence the color of the flour and, indirectly, the color of the finite products [42]. 

The texture parameters are interdependent of the mouthfeel of bread. For all optimal breads, a more desirable firmness was observed in comparison with wheat bread. The obtained results can be explained by the lower content of starch and higher content of fiber from quinoa flour, which could restrain starch retrogradation. This phenomenon involves the reorganisation of starch component molecules into an ordered structure. The rate of retrogradation is influenced by the availability of water, since the crystallization of amylopectin needs to incorporate water molecules into crystallites [43].

An improvement of the nutritional profile was also obtained in the case of optimal wheat–quinoa composite flour bread related to the quinoa flour particle sizes. The protein and lipid content increased considerably for all optimal bread samples compared to the wheat flour bread. This improvement can be explained by the high content of proteins and lipids, especially in the medium-sized particles of quinoa flour [44]. Similar results regarding the nutritional composition of wheat–quinoa composite bread were obtained by other authors [15,45], while studies on the variation of nutrients in wheat flour bread with the addition of quinoa flour of different particle sizes are lacking.

The increase in mineral content in the optimal bread formulated with quinoa flour could be due to higher mineral content in quinoa seeds compared to wheat. These results are also in agreement with those reported by El-Sohaimy et al. [46], who found a higher content of minerals (Fe, Ca and Zn) in pan bread supplemented with quinoa flour; however, no study on mineral content has been carried out, until now, on bread containing different quinoa flour particle sizes. The results of the present research could be useful in the treatment of mineral deficiencies (especially iron, calcium and zinc) that have a negative effect on human health and could lead to iron deficiency anemia, rickets, osteoporosis and diseases of the immune system [47]. A similar mineral content of quinoa flour bread in doses of 10% was also reported by other researchers [15,16,46].

The increase in essential amino acid content in the optimal bread corresponding to study particle sizes may be due to the high content of these amino acids in quinoa seeds, which have a higher essential amino acid content than wheat protein [46]. Until now, no research has been carried out on the amino acid content of bread obtained from wheat–quinoa composite flour, for different particle sizes.

Replacing wheat flour with different quinoa particle sizes at typical doses had a significant effect on the sensory quality of bread, i.e., the taste, smell, crumb structure, appearance, and overall acceptability. Our results fall in line with other research studies that highlight that bread with 5 and 20% quinoa flour proportion had an overall acceptable sensory quality [48,49].

### 3.4. Relationships between Dynamic Dough Rheological Parameters, and Bread Physical-Chemical, Textural and Sensory Characteristics

The Pearson correlation analysis revealed significant correlations (*p* < 0.05) between the evaluated parameters. Very strong correlations (0.95 < r < 0.99) were obtained between the dough rheological parameters and the chemical constituents, physical parameters, texture and sensory characteristics of the bread. Positive correlations were found between the protein content and elastic and viscous moduli of dough (r = 0.99 and r = 0.96, respectively), but protein was negatively correlated with bread firmness (r = −0.96), while the bread lipids negatively influenced only its cohesiveness (r = −0.95). An indirect relationship was found between the viscosity factor and the lipid content of the bread (r = −0.96). The formation of amylose–lipid complexes alters the gelatinization characteristics of starch [50,51]. Bread firmness was negatively associated with bread appearance (r = −0.95), while bread elasticity was positively influenced by cohesiveness (r = 0.99), viscosity factor (r = 0.97) and maximum gelatinization temperature (r = 0.99). Bread volume was negatively correlated with viscosity factor (r = −0.90) and bread taste (r = −0.97), and bread porosity with maximum gelatinization temperature (r = −0.99). The bread appearance was positively influenced by the elastic and viscous moduli (r = 0.98 and r = 0.96, respectively). These correlations between dough rheological parameters and the chemical constituents of bread support the findings of some researchers [35,52,53].

By using the principal component analysis (PCA), the relationships between the assessed parameters and the type of the sample were highlighted on the two principal components of the bi-plot. The first principal component (PC1) was associated with the dynamic dough rheological parameters (G′, G″, tan δ, T_max_ and Jr_max_), with the protein and lipid content of the bread, the bread textural parameters (firmness, elasticity, cohesiveness), physical properties (volume and porosity), and the sensory characteristics, appearance and taste. The second principal component (PC2) was associated with maximum creep compliance, bread moisture and carbohydrate content, bread chewiness, and overall bread acceptability.

## 4. Materials and Methods

### 4.1. Materials

The materials used were wheat flour (*Triticum aestivum*) (ash content max. 0.65%) (Mopan, Suceava, România), fresh yeast (Rompak, Paşcani, România), salt, quinoa seeds (SanoVita, Vâlcea, România) and water. Quinoa seeds (*Chenopodium quinoa* Willd.) of the following composition: protein (14.12%), lipids (5.61%), and ash (2.00%), reported to dried substances, were used as an enrichment ingredient. Quinoa seeds purchased from the local supermarket (Suceava, România) were milled with a laboratory ultra-centrifugal mill (Grain Mill, KitchenAid, Model 5KGM, Whirlpool Corporation, Benton Harbor, MI, USA) and sieved with a Retsch Vibratory Sieve Shaker AS 200 basic (Haan, Germany) for half hour at 70 Hz amplitude to obtain the following quinoa flour particle sizes: large, QL (>300, <500 µm), medium, QM (>180, <300 µm) and small, QS (<180 µm) which then were packed into sealed zip plastic bags and stored at 4 ± 1 °C for further use. 

### 4.2. Optimal Composite Flour Preparation 

The wheat–quinoa composite flour samples (OF_QL, OF_QM, and OF_QS) were prepared by adding the optimal doses of 9.13, 10.57, and 10.25% quinoa flour—established for large, medium and small particle size, respectively, in a previous study [54]—to wheat flour, and mixing for 30 min in a Yucebas Y21 machine (Izmir, Turkey). Wheat flour was considered the control.

### 4.3. Dough and Bread-Making Preparation

The dough was obtained from 300 g of flour, 12 g of yeast, 5.4 g of salt, and water until the dough reach the optimum consistency determined at Mixolab (Chopin, Tripetteet Renaud, Paris, France) [44]. When the dough was prepared for empirical and fundamental tests, the yeast was not used. The yeast was only used for the rheofermentometer and texture analysis. The indirect method of dough preparation was carried out by mixing half flour with all water and yeast in order to form a leaven at 30 ± 2 °C and 85% relative air humidity (RH) and leaving this mixture for two hours in a fermentation chamber (PL2008, Piron, Cadoneghe, Padova, Italy). When fermentation finished, the second part of the flour with the salt was added, mixed for 10 min using a Kitchen Aid mixer (Whirlpool Corporation, Benton Harbor, MI, USA), and allowed to complete its process of sugar fermentation for another hour in the same conditions [54,55,56]. After that, portions of 400 were shaped manually, placed in molds and subjected to final fermentation for 60 min. The bread was baked in a Caboto PF8004D (Cadoneghe, Padova, Italy) at 220 ± 5 °C, for 25 ± 2 min. Additionally, the control bread was made from wheat flour (WFB). The loaves were subsequently analyzed after being cooled at room temperature for two hours.

### 4.4. Proximate Analysis of Wheat Flour, Optimal Composite Flour, and Optimal Bread Samples

The flours were analyzed according to the International Association for Cereal Chemistry standard methods [57]; the moisture content was analyzed based on gravimetric method (110/1), and the protein by the Kjeldahl method as described by the ICC 105/2 method. It was calculated with a general factor of 6.25 for wheat flour and bread, and 5.53 for optimal wheat–quinoa composite flour and bread. Lipids were analyzed by acid hydrolysis and the solvent extraction method as described by ICC 136, and ash content was produced by incineration at 900 °C for 8 h in a muffle furnace (Nabertherm, LE 2/11/R6, Bremen, Germany) until light gray ash was obtained (ICC 104/1). The content of total carbohydrates was calculated by subtracting the total contents of moisture, total ash, crude protein, and crude fat from 100% of dry matter. The gross energy content was determined by calculation from crude fat, carbohydrate, and crude protein contents using Atwater’s conversion factors: 4.1 kcal per g for protein and carbohydrates, and 9.3 kcal per g for fats [58]. Each analysis was conducted at least in duplicate.

### 4.5. Mineral Content Determination by Atomic Absorption Spectrometry

The K, Ca, Mg, Na, Zn, Fe, Mn, and Cu elements contents of the wheat flour, optimal wheat–quinoa composite flour and bread were determined by flame atomic absorption spectrometry (FAAS) (AA-6300 Shimadzu, Kyoto, Japan). The analysis of the sample involved two stages: the mineralization of the sample and the metal dosage by spectrophotometry. During mineralization, the organic matter from the sample (5.00 ± 0.001 g) is destroyed by carbonization and combustion in a muffle furnace (Nabertherm, LE 2/11/R6, Bremen, Germany), with the temperature gradually increasing from 250 to 450 °C, up to 900 °C, for 8 h. 5 mL HCl 6 mol/L (STAS 13013/1-91) is added to the ash obtained, and then the acid is evaporated using a sand bath, and the residue is dissolved with 730 µL HNO3 69% and brought to the mark (50 mL) with deionized water. Deionized water was used as a control sample, following the same procedure. The spectrophotometric determination involved the following steps: activating the hollow cathode lamp corresponding to the elements (K, Ca, Mg, Na, Fe, Zn, Mn, Cu), adjusting the operational parameters (wavelength, sensitivity), activating and adjusting the flame, as well as establishing the curve standard by absorbing four working standard solutions of different concentrations. The calibration curve made for each element covers the range of 0.5–5.0 mg/L Ca, 0.5–2.5 mg/L Cu, 0.5–5.0 mg/L Fe, 0.05–0.30 mg/L Mg, 0.5–3.0 mg/L Mn, 0.05–0.60 mg/L Zn, 0.1–0.5 mg/L Na, and 0.2–1.0 mg/L K. The wavelengths taken into account when determining Ca, Cu, Fe, Mg, Mn, Zn, K and Na elements correspond to 422.7, 342.7, 248.3, 285.2, 279.5, 213.8, 589.0, and 766.5 nm. Air-acetylene as the flame type, a gas flow rate of 15.0 L/min, a pre-spray time of 10 s, an integration time of 5 s, and a response time of 1 s were also included as working conditions. The mineral elements are expressed as mg/100 g of flour and were calculated with Equation (1):

(1) $$E=\frac{C\cdot F\cdot V}{M}$$

where: E—Mineral element concentration, mg/100 g; C—The concentration measured on the calibration curve, mg/L; F—Dilution factor; V—Sample volume, mL; M—Sample mass taken in the analysis, g.

### 4.6. Amino Acid Content Determination of Wheat Flour, Optimal Wheat–Quinoa Composite Flour, and Optimal Bread Samples

For the extraction and quantification of the amino acids from the wheat flour, the optimal wheat–quinoa composite flour, and the bread, 3.70 ± 0.1 g of sample was mixed with 30 mL of 15% trichloroacetic acid (TCA). The pH of the solution was adjusted to 2.2 with sodium hydroxide solution and diluted with 50 mL of 15% TCA [59]. Then, the supernatant was collected and filtered through a 0.45-µm microfilters, and 100 µL supernatant was subjected to the determination of organic components using the EZ:faast GC-MS kit (Phenomenex, Torrance, CA, USA). A Shimadzu GC/MS system (GC MS-QP 2010 Plus, Shimadzu, Kyoto, Japan) with a Zebron ZB-AAA GC column (10 m × 0.25 mm) was employed to determine the amino acids. The analysis time was 10 min and the injected volume was set to 0.002 mL. The split-less injection mode was applied. The initial temperature of the GC oven was 110 °C, which was increased to 320 °C and held for three min. The temperature conditions used for the mass spectrometer were 200 °C for the ion source and 320 °C for the interface. The quadrupole measured ion abundances from 35 to 500 m/z. Amino acid mixture solutions included in the kit were used for calibration [60].

### 4.7. Dough Rheological Analysis

#### 4.7.1. Dynamic Rheological Tests

Dough dynamic rheological properties were assessed by using a Thermo-HAAKE, MARS 40 (Karlsruhe, Germany) with parallel plate–plate geometry. The dynamic parameters measured were elastic modulus (G′), viscous modulus (G″), viscosity factor (tan δ), maximum gelatinization temperature (T_max_), and creep-recovery compliance (Jc_max_, Jr_max_).

#### 4.7.2. Empirical Rheological Tests

The Falling number index (FN) of the wheat flour and optimal wheat–quinoa composite flour related to each studied particle size of QF was determined using a Falling number device (FN 1305, Perten Instruments AB, Stockholm, Sweden) in order to determine the amylolytic activity.

Mixolab Chopin equipment (Tripette et Renaud, Paris, France) was used for a complete rheological test of wheat and optimal wheat–quinoa composite flour following the ICC 173, AACC 54-60.01 method [57]. The Mixolab parameters of water absorption, WA (%), dough development time, DT (min), the stability of the dough, ST (min), protein weakening (C1-2), starch gelatinization (C3-2), starch breakdown (C3-4), and starch recrystallization (C5-4) were determined.

The rheological properties during dough biaxial extension, tenacity (P), extensibility (L), dough strength (W), and alveograpic ratio (P/L) were determined with Alveograph Chopin equipment (Villeneuve-la-Garenne, France) following the ICC 121 method [57]. 

The fermentation test of dough was performed with the rheofermentometer device (Chopin Rheo, type F4, Villeneuve-La-Garenne, France) following the AACC 89–01.01 method [61]. The determined fermentation parameters were the maximum height of the gas release curve (H′m), the total volume of CO_2_ production (VT), the volume of the gas retained in the dough at the end of the test (VR), and the retention coefficient (CR).

### 4.8. Bread Physical and Textural Characteristics Determination

The physical characteristics of the bread samples made from optimal wheat–quinoa composite flour and wheat flour, bread volume (BV), specific volume (Sp_Volume), porosity, and elasticity were determined according to the Romanian standard SR 90: 2007 [62]. A TVT-6700 texture analyzer (Perten Instruments, Hägersten, Sweden) was used for analyzing bread firmness, springiness, cohesiveness, gumminess, resilience and masticability using the working setting shown in a previous study [54], and the values were recorded and processed by TexCalc 5 software (5.1.0.x. version, Perten Instruments, Hägersten, Sweden). 

### 4.9. Bread Color Measurement 

The procedure for bread color assessment is based on the determination of the CIE L*, a*, b* values, when L* indicates the lightness, a*, chromaticity on a green (−) to red (+), and b*, chromaticity on a blue (−) to yellow (+). Crumb and crust color analysis was performed at room temperature according to the reflectance method with a CR-700 colorimeter (Konica Minolta Inc., Tokyo, Japan), by measuring at five different points on the surface, respectively, in the center of the middle slice of bread. The parameters of the colorimeter in the reflection mode were set as follows: a standard illuminant D65, observation angle of 10°, and an aperture of 30 mm. The total color differences (ΔE) were calculated according to the methods of [63], with Equation (2):

(2) $$\Delta E=\sqrt{{\left(L_{1}^{*}-L_{0}^{*}\right)}^{2}+{\left(a_{1}^{*}-a_{0}^{*}\right)}^{2}+{\left(b_{1}^{*}-b_{0}^{*}\right)}^{2}}$$

where $L_{0}^{*}$, $a_{0}^{*}$, and $b_{0}^{*}$ are the color values of the white flour bread and $L_{1}^{*}$, $a_{1}^{*}$, and $b_{1}^{*}$, are the experimental color values of the different optimal quinoa breads.

### 4.10. Sensory Evaluation of Breads

The evaluation of bread sensory attributes was performed in accordance with the Romanian Standard SR ISO 11035:2007 [63]. The 13 semi-trained panelists aged between 20–55 years were instructed to evaluate the coded samples for overall acceptability, appearance, crust and crumb structure, taste, and smell using a hedonic scale (1–9) [64]. A score of 1 represented “extreme dislike”, a score of 5, “neither like nor dislike” and a score of 9 represented “extreme like”. The panelists’ acuity was previously tested according to international standards ISO 8586-1:2012, ISO 8586-2:2014, and ISO 3972 in a sensory lab at Stefan cel Mare University. The panelists were instructed to rinse their mouths with water to clean the palate between evaluations.

### 4.11. Statistical Analysis

The data were shown as mean with standard deviation, and for every sample, the experiments were conducted at least in duplicate. SPSS software 25.0 (trial version) (IBM, New York, NY, USA) was employed to conduct the statistical analysis of the data. Statistically significant differences between samples were determined by a one-way ANOVA. The relationships among the studied parameters were tested by using Pearson’s coefficient at *p* < 0.05. A principal component analysis (PCA) was applied to highlight the relationships between the wheat–quinoa flour dough, bread proximate composition, textural characteristics and acceptability, and to observe similarities or dissimilarities between them. 

## 5. Conclusions

Compared to the wheat bread, the physical characteristics of the bread made from optimal wheat–quinoa composite flour showed an improvement in volume, porosity, and elasticity, and also in firmness, springiness, and chewiness for all particle sizes, while the lightness of the bread crust and crumb decreased. The highest values for volume, porosity and crumb elasticity were found for optimal bread with medium quinoa particle sizes (OB_QM), while firmness decreased, indicating an improvement for this bread. The nutritional composition of optimal bread revealed a considerable increase in protein, lipid, ash, total amino acids, and mineral content, whereas carbohydrates decreased compared to wheat bread. The optimal bread with medium particles presented the highest values for nutritional composition, while the energy value was the lowest. The mineral content was up to 2.3 times higher in the optimal bread compared to wheat flour bread; the highest value registered was from bread with a medium particle size. The essential amino acid content followed the same trend and represented up to 14.80% of the total amino acid content; the highest value was registered for bread with a medium particle size, which also presented the highest protein content. Sensory evaluation analysis showed that optimal bread with medium and large particles of quinoa flour exhibited an increase in scores for overall acceptability, appearance, crumb structure, taste, and smell. Of all optimal breads, the bread with medium particles, presenting a score higher than 8.8 for the general acceptability, was the one very well accepted. 

Considering the nutritional improvement and the consumers’ acceptance of bread with medium quinoa particle size, we can conclude that this optimal bread is the most suitable for the needs of the market. This study demonstrated that the partial substitution of wheat bread with quinoa flour of different particle sizes at typical doses has the potential to improve the physical textural, nutritional, and sensorial properties of formulated bread.

## Figures and Tables

**Table 1:** The mineral content of the optimal wheat–quinoa composite flour corresponding to large, medium, and small size particles sizes of quinoa flour versus wheat flour.

Elements (mg/100 g)	WF	QF	Particle Size		
			OF_QL	OF_QM	OF_QS
K	108.50 ± 2.12 ^c^	243.50 ± 10.60 ^a^	135.70 ± 10.05 ^b^	127.29 ± 21.21 ^bc^	116.00 ± 8.13 ^bc^
Ca	24.80 ± 0.10 ^c^	51.00 ± 0.02 ^a^	26.44 ± 0.30 ^b^	27.71 ± 0.48 ^b^	27.77 ± 0.48 ^b^
Mg	155.50 ± 0.65 ^b^	166.90 ± 0.24 ^a^	155.81 ± 1.05 ^b^	156.83 ± 0.14 ^b^	156.92 ± 0.14 ^b^
Na	7.33 ± 0.11 ^c^	12.49 ± 0.03 ^a^	7.76 ± 1.20 ^bc^	7.84 ± 0.62 ^b^	7.81 ± 0.44 ^b^
Fe	1.80 ± 0.06 ^c^	8.12 ± 0.13 ^a^	2.31 ± 0.04 ^bc^	2.43 ± 0.86 ^b^	2.61 ± 0.12 ^b^
Zn	3.02 ± 0.25 ^a^	4.07 ± 0.03 ^a^	3.34 ± 0.32 ^a^	3.43 ± 0.08 ^a^	3.44 ± 0.13 ^a^
Mn	1.59 ± 0.10 ^b^	7.00 ± 0.02 ^a^	1.70 ± 0.69 ^b^	1.90 ± 0.37 ^b^	1.87 ± 0.02 ^b^
Cu	0.56 ± 0.01 ^c^	1.52 ± 0.06 ^a^	0.64 ± 0.04 ^bc^	0.66 ± 0.12 ^b^	0.67 ± 0.10 ^b^

WF—Wheat flour; QF—Quinoa flour; OF_QL—Optimal composite flour with large quinoa particle size; OF_QM—Optimal composite flour with medium quinoa particle size; OF_QS—Optimal composite flour with small quinoa particle size. Mean values on the same row followed by different letters (a–c) are significantly different (*p* < 0.05).

**Table 2:** The characteristics of the optimal wheat–quinoa composite flour for each quinoa flour particle size and dough rheological parameters.

Parameters	WF	OF_QL	OF_QM	OF_QS
FN (s)	312.00 ± 5.25^axA^	305.36 ± 5.25 ^a^	305.50 ± 5.25 ^x^	315.25 ± 57.45 ^A^
**Mixolab**				
WA (%)	58.50 ± 0.02 ^axA^	57.72 ± 0.25 ^a^	58.25 ± 0.54 ^x^	59.20 ± 2.15 ^A^
DT (min)	1.69 ± 0.75 ^axA^	2.48 ± 0.75 ^b^	2.21 ± 0.98 ^y^	2.80 ± 1.60 ^B^
ST (min)	9.96 ± 0.65 ^axA^	9.25 ± 0.66 ^a^	9.80 ± 0.05 ^x^	9.75 ± 0.52 ^A^
C1-2 (N m)	0.61 ± 0.02 ^axA^	0.58 ± 0.25 ^a^	0.60 ± 0.02 ^x^	0.62 ± 0.01 ^A^
C3-2 (N∙m)	1.41 ± 0.03 ^axA^	1.38 ± 0.02 ^a^	1.40 ± 0.01 ^x^	1.25 ± 0.01 ^A^
C3-4 (N∙m)	0.05 ± 0.04 ^axA^	0.18 ± 0.01 ^b^	0.09 ± 0.01 ^x^	0.10 ± 0.10 ^B^
C5-4 (N∙m)	1.15 ± 0.01 ^bxA^	0.94 ± 0.01 ^a^	0.90 ± 0.05 ^y^	0.90 ± 0.04 ^B^
**Alveograph**				
P (mm H_2_O)	87.00 ± 5.75 ^axA^	99.51 ± 5.20 ^b^	100.50 ± 5.25 ^y^	92.15 ± 5.30 ^B^
L (mm)	91.00 ± 10.50 ^byB^	47.07 ± 5.21 ^a^	37.89 ± 5.40 ^x^	55.45 ± 2.45 ^A^
W (10^−4^ J)	253.00 ± 20.14 ^byB^	177.93 ± 10.95 ^a^	170.52 ± 20.45 ^x^	185.75 ± 25.41 ^A^
P/L (adim.)	0.95 ± 0.05 ^axA^	2.23 ± 00.20 ^b^	3.20 ± 0.05 ^y^	1.87 ± 0.52 ^B^
**Rheofermentometer**				
H’_m_ (mm)	62.00 ± 4.25 ^axA^	64.48 ± 0.05 ^b^	64.25 ± 2.50 ^x^	65.20 ± 0.01 ^B^
VT (mL)	1168.00 ± 89.56 ^axA^	1249.36 ± 22.11 ^b^	1205.02 ± 12.20 ^y^	1200.25 ± 21.25 ^A^
VR (mL)	991.20 ± 85.25 ^axA^	1081.93 ± 9.50 ^b^	990.45 ± 75.70 ^x^	1000.15 ± 22.31 ^A^
CR (%)	84.20 ± 2.50 ^axA^	84.69 ± 2.85 ^a^	83.75 ± 2.95 ^x^	85.95 ± 2.45 ^A^
**Rheometer**				
G′ (Pa)	26,370.00 ± 10.00 ^bxA^	43,560.83 ± 257.50 ^b^	47,856.52 ± 470.25 ^y^	47,520.25 ± 252.85 ^B^
G″ (Pa)	9488.00 ± 74.58 ^bxA^	14,573.01 ± 145.45 ^b^	13,560.54 ± 129.45 ^y^	12,900.55 ± 258.25 ^B^
tan δ (adim.)	0.3610 ± 0.02 ^ayB^	0.3312 ± 0.01 ^a^	0.3302 ± 0.01 ^x^	0.3301 ± 0.01 ^A^
T_max_ (°C)	83.24 ± 0.55 ^byB^	79.16 ± 0.96 ^a^	78.25 ± 0.95 ^x^	79.20 ± 0.20 ^A^
Jc_max_ (10^−5^ Pa^−1^)	24.50 ± 4.50 ^ayA^	17.71 ± 2.51 ^a^	18.25 ± 0.85 ^x^	24.52 ± 4.52 ^A^
Jr_max_ (10^−5^ Pa^−1^)	16.62 ± 2.40 ^ayA^	12.45 ± 0.58 ^a^	12.75 ± 3.20 ^x^	17.58 ± 2.25 ^A^

WF—Wheat flour; OF_QL—Optimal composite flour with large quinoa particle size; OF_QM—Optimal composite flour with medium quinoa particle size; OF_QS—Optimal composite flour with small quinoa particle size. Mean values on the same row followed by different letters are significantly different (*p* < 0.05): a, b (OF_QL), x, y (OF_QM), and A, B (OF_QS), respectively, for differences between the control and optimal formulation values. FN—Falling number; WA—Water absorption capacity; DT—Development time; ST—Dough stability; C1–2—Protein denaturation; C3–2—Starch gelatinization; C3–4—Stability of hot starch gel; C5–4—Starch retrogradation; P—Tenacity; L—Extensibility; W—Deformation energy; P/L—Alveographic ratio; H’m—Maximum height; VT—Total volume of gas; VR—Volume of gas retained; CR—Gas retention coefficient; G′—Elastic modulus; G″—Viscous modulus; tan δ—Viscosity factor; T_max_ —Maximum gelatinization temperature; Jc_max_—Maximum creep compliance; Jr_max_ —Maximum recovery compliance.

**Table 3:** Physical parameters of breads with the optimal dose corresponding to large, medium and small particle size of quinoa flour versus wheat flour bread.

Bread Sample	Volume (cm^3^)	Specific Volume (cm^3^/g)	Porosity (%)	Elasticity (%)
WFB	352.20 ± 15.25 ^d^	2.45 ± 0.25 ^c^	64.22 ± 5.62 ^d^	91.70 ± 6.52 ^d^
OB_QL	407.90 ± 25.89 ^a^	3.22 ± 0.25 ^a^	73.92 ± 6.35 ^a^	95.83 ± 5.45 ^a^
OB_QM	391.91 ± 25.50 ^c^	2.81 ± 0.62 ^c^	70.54 ± 5.75 ^c^	95.14 ± 8.55 ^c^
OB_QS	392.82 ± 25.45 ^b^	2.82 ± 0.71 ^b^	72.64 ± 4.78 ^b^	95.72 ± 4.65 ^b^

WFB—Wheat flour bread; OB_QL—Optimal bread with large quinoa particle size; OB_QM—Optimal bread with medium quinoa particle size; OB_QS—Optimal bread with small quinoa flour particle size. Mean values on the same column followed by different letters are significantly different (*p* < 0.05).

**Table 4:** Color parameters of breads with the optimal dose corresponding to large, medium and small particle size of quinoa flour vs. wheat flour bread.

Bread Sample	Crust Color Parameters				Crumb Color Parameters			
	L*	a*	b*	ΔE	L*	a*	b*	ΔE
WFB	70.35 ± 0.91 ^a^	−1.33 ± 0.22 ^c^	32.27 ± 0.28 ^a^	-	73.94 ± 0.27 ^a^	−4.48 ± 0.03 ^a^	20.02 ± 0.23 ^ab^	-
OB_QL	69.08 ± 0.53 ^a^	1.00 ± 0.77 ^b^	33.21 ± 1.46 ^a^	2.73 ± 0.05 ^b^	66.89 ± 1.46 ^b^	−4.13 ± 0.16 ^a^	19.49 ± 0.52 ^b^	9.34 ± 0.75 ^b^
OB_QM	64.75 ± 0.40 ^b^	2.65 ± 0.10 ^a^	34.20 ± 0.43 ^a^	7.78 ± 1.15 ^ab^	63.72 ± 1.12 ^b^	−3.83 ± 0.07 ^a^	20.73 ± 0.64 ^ab^	13.12 ± 1.21 ^ab^
OB_QS	61.17 ± 0.84 ^c^	2.21 ± 0.27 ^a^	32.41 ± 0.96 ^a^	10.25 ± 1.02 ^a^	65.54 ± 1.02 ^b^	−3.93 ± 0.16 ^a^	21.14 ± 0.47 ^a^	13.29 ± 0.95 ^a^

WFB—Wheat flour bread; OB_QL—Optimal bread with large quinoa particle size; OB_QM—Optimal bread with medium quinoa particle size; OB_QS—Optimal bread with small quinoa flour particle size. L*, a*, b*, ΔE—The degree of lightness, the intensity of red or green, respectively of yellow or blue, and delta E, the total color differences. Mean values on the same column followed by different letters are significantly different (*p* < 0.05).

**Table 5:** Texture parameters of bread with the optimal dose corresponding to the large, medium and small particle size of quinoa flour vs. wheat flour bread.

Bread Sample	Firmness (N)	Springiness (Adim.)	Cohesiveness (Adim.)	Gumminess (N)	Resilience (Adim.)	Masticability (N)
WFB	5.71 ± 0.02 ^a^	1.3457 ± 0.27 ^a^	0.8575 ± 0.01 ^a^	499.73 ± 4.63 ^a^	1.8278 ± 0.00 ^ab^	499.73 ± 4.63 ^a^
OB_QL	4.74 ± 0.11 ^b^	1.0000 ± 0.00 ^b^	0.8629 ± 0.03 ^a^	179.74 ± 14.60 ^b^	1.6117 ± 0.09 ^b^	179.74 ± 14.60 ^b^
OB_QM	4.43 ± 0.06 ^c^	1.0008 ± 0.00 ^b^	0.8731 ± 0.02 ^a^	205.76 ± 20.22 ^b^	1.9724 ± 0.04 ^a^	205.76 ± 20.22 ^b^
OB_QS	4.39 ± 0.09 ^d^	1.0009 ± 0.00 ^b^	0.8690 ± 0.01 ^a^	173.13 ± 14.83 ^b^	2.0003 ± 0.19 ^a^	173.13 ± 14.83 ^b^

WFB—Wheat flour bread; OB_QL—Optimal bread with large quinoa particle size; OB_QM—Optimal bread with medium quinoa particle size; OB_QS—Optimal bread with small quinoa flour particle size. Mean values on the same column followed by different letters are significantly different (*p* < 0.05).

**Table 6:** Physico-chemical characteristics of bread with the optimal dose corresponding to large, medium, and small particle size of quinoa flour vs. wheat flour bread.

Bread Sample	Moisture (%)	Proteins (%)	Lipids (%)	Ash (%)	Carbohydrates (%)	Energetic Value (kcal)
WFB	43.12 ± 0.03 ^bc^	8.35 ± 0.13 ^c^	0.01 ± 0.00 ^c^	0.72 ± 0.02 ^b^	47.81 ± 0.11 ^a^	230.31 ± 0.22 ^b^
OB_QL	43.43 ± 0.03 ^b^	9.01 ± 0.12 ^bc^	0.09 ± 0.03 ^bc^	0.75 ± 0.02 ^b^	47.02 ± 0.04 ^a^	232.10 ± 0.08 ^a^
OB_QM	44.21 ± 0.02 ^a^	9.96 ± 0.06 ^a^	0.15 ± 0.02 ^a^	0.82 ± 0.02 ^a^	44.86 ± 0.13 ^ab^	227.38 ± 0.05 ^cd^
OB_QS	43.02 ± 0.14 ^c^	9.93 ± 0.13 ^b^	0.13 ± 0.02 ^b^	0.74 ± 0.03 ^b^	46.17 ± 0.03 ^c^	231.31 ± 0.57 ^ab^

WFB—Wheat flour bread; OB_QL—Optimal bread with large quinoa particle size; OB_QM—Optimal bread with medium quinoa particle size; OB_QS—Optimal bread with small quinoa flour particle size. Mean values on the same column followed by different letters are significantly different (*p* < 0.05).

## Data Availability

Data is contained within the article.

## Supplementary Materials

The following supporting information can be downloaded at: https://www.mdpi.com/article/10.3390/plants12040698/s1, Figure S1: The appearance and section of the bread with the optimal dose of quinoa flour corresponding to the large, medium, and small particle size (OB_QL, OB_QM, and OB_QS) compared to wheat flour bread (WFB).
