# Supplementary material for: Nutritionally Improved Wheat Bread Supplemented with Quinoa Flour of Large, Medium and Small Particle Sizes at Typical Doses
Source: Plants (Basel). 2023 Feb 4;12(4):698. doi: 10.3390/plants12040698 (PMC9964393; doi:10.3390/plants12040698)
Supplement: Supplementary file 1 [file plants-12-00698-s001.zip › plants-2176349-supplementary.pdf]

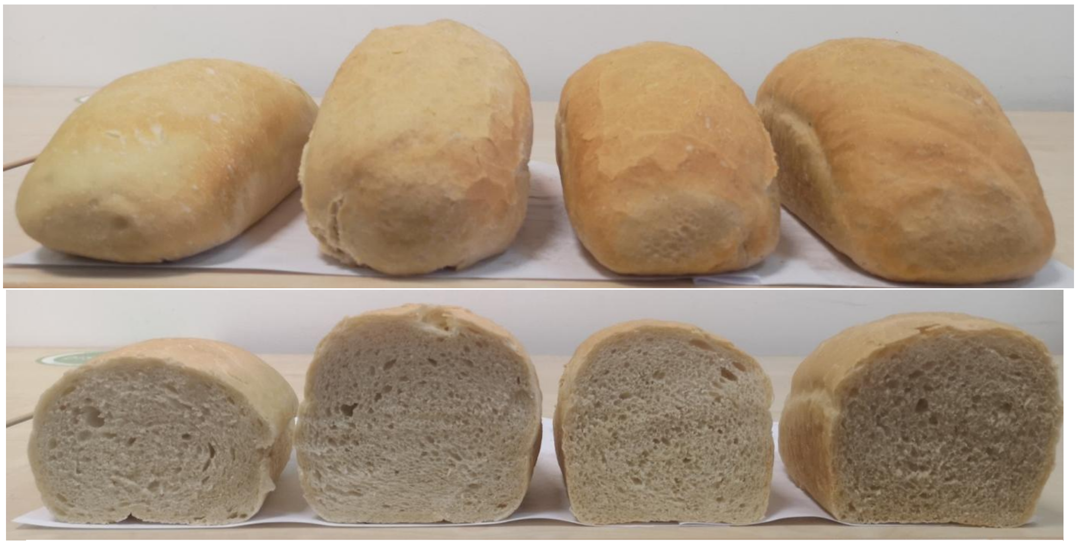

WFB OB_QL OB_QM OB_QS

Figure S1. The appearance and section of the bread with the optimal dose of quinoa flour corresponding to the large, medium, and small particle size (OB_QL, OB_QM, and OB_QS) compared to wheat flour bread (WFB).
